# Mesoporous Materials as Elements of Modern Drug Delivery Systems for Anti-Inflammatory Agents: A Review of Recent Achievements

**DOI:** 10.3390/pharmaceutics14081542

**Published:** 2022-07-25

**Authors:** Michał Moritz, Małgorzata Geszke-Moritz

**Affiliations:** 1 Department of Pharmaceutical Chemistry, Pomeranian Medical University in Szczecin, Plac Polskiego Czerwonego Krzyża 1, 71-251 Szczecin, Poland; 2 Department of Pharmacognosy and Natural Medicines, Pomeranian Medical University in Szczecin, Plac Polskiego Czerwonego Krzyża 1, 71-251 Szczecin, Poland

**Keywords:** anti-inflammatory drugs, mesoporous materials, drug delivery systems, controlled release, physicochemical properties

## Abstract

Interest in the use of mesoporous materials as carriers of medicinal substances has been steadily increasing in the last two decades. Mesoporous carriers have application in the preparation of delivery systems for drugs from various therapeutic groups; however, their use as the carriers of anti-inflammatory agents is particularly marked. This review article, with about 170 references, summarizes the achievements in the application of mesoporous materials as the carriers of anti-inflammatory agents in recent years. This article will discuss a variety of mesoporous carriers as well as the characteristics of their porous structure that determine further use of these materials in the field of medical applications. Special attention will be paid to the progress observed in the construction of stimuli-responsive drug carriers and systems providing site-specific drug delivery. Subsequently, a review of the literature devoted to the use of mesoporous matrices as the carriers of anti-inflammatory drugs was carried out.

## 1. Introduction

According to the IUPAC nomenclature, mesoporous materials are defined as materials with a pore size between 2 and 50 nm [1]. Extraordinary properties of mesoporous materials make them attractive tools in many fields of science including heterogeneous catalysis [2], photocatalysis [3], hydrogen production [4], electronics [5], solar cells [6] or battery components [7]. These materials are of particular interest in the field of medicine and pharmacy. Mesoporous matrices have attracted increasing attention in drug delivery [8], bioanalysis [9], cell and tissue imaging [10], biomolecule separation [11] and tissue engineering [12]. 

In the last two decades, the use of mesoporous materials as the carriers in drug delivery systems has given scientists almost unlimited possibilities to modify the release of active substances from various therapeutic groups [13]. Mesoporous matrices allow incorporation of numerous classes of drug molecules. They have been shown to store and gradually release therapeutically relevant agents like antibiotics [14], anti-virus drugs [15], anti-cancer drugs [16], vitamins [17], anti-hypertensive drugs [18], lipid-lowering agents [19], anti-fungal drugs [20], antibacterial agents [21], anti-ulcer drugs [22], anti-osteoporosis drugs [23], anti-platelet drugs [24], cardiac drugs [25] and choleretic drugs [26] among others. Mesoporous silica carriers are particularly useful for drug molecules characterized by low solubility and a lack of specificity. Scientists were greatly interested in the use of mesoporous carriers for drugs with anti-inflammatory activity. Numerous anti-inflammatory drugs are poorly water-soluble. Using these medications may lead patients to take high doses of the drug to achieve sufficient therapeutic effect; this is the main cause of adverse drug effects, particularly for drugs with a narrow therapeutic window [27]. Drug delivery systems enable to deliver the specific dose of the drug to the target site in the body while ensuring controlled rate and drug release time to maintain its therapeutic concentration for a long time. Modern stimuli-responsive drug delivery systems ensure controlled release of active substance that depends on the pH [28], temperature [29], light [30], ultrasounds [31], osmotic conditions [32], oxidation state [33] or the presence of enzymes [34] among others. 

Mesoporous materials are extremely attractive drug reservoirs due to their physicochemical properties such as high specific surface area, pore ordering, tunable pore size, large pore volume and pore diameter as well as the possibility of surface modification using various chemical functions [35]. Moreover, depending on their chemical structure, mesoporous carriers may exhibit magnetic [36], conducting [37] or fluorescent [38] properties. This group of materials includes substances of different chemical classes, namely carbon, silica, metals, metal salts, metal hydroxides, alkaline and amphoteric metal oxides, transition metal oxides, lanthanide oxides, perovskite-type/spinel-type oxides, organic structures, nitrides/carbides and other hybrid materials that were widely studied in recent years [35]. 

With the development of the highly ordered mesoporous silica MCM-41 (Mobile Composition of Matter) in 1992, mesoporous materials have become an intense research focus [39]. MCM-41 mesoporous silica possessing organized mesoporous structure, a high degree of porosity, narrow pore size distribution, large surface area, hexagonally formed pores, controllable pore volume and biocompatibility has attracted great research interest as a carrier in drug delivery systems (DDSs) [40]. The exceptional structure of mesoporous materials enables the efficient loading of drugs and their subsequent controlled release. 

Mesoporous silicas have been demonstrated to be able to incorporate high dosages of drugs in the internal pore system. The properties that influence the process of drug loading and drug release are specific surface areas, pore volumes, pore diameters, pore arrangement, pore channel length as well as particle size and morphology [41,42,43]. The properties of siliceous material pores (mainly pore size and pore volume) as well as the connectivity and geometry that determine the degree of drug loading and drug release are summarized in Table 1. 

It has been demonstrated that as the pore size decreases, the amount of drug loaded is reduced. The drug release rate also decreases as a result of increasingly tightly packed molecules in the mesopores [50]. Some data supports the existence of a critical pore width which exerts steric hindrance to drug diffusion through the pores [56]. It has been shown that the release rate of drugs characterized by higher molecular weight is decreased as compared to ones with lower molecular weight from the pores of the same diameter [57]. Some authors claim that to allow for easy access of drug for loading and release, the pore size should be at least three times greater than the drug molecule diameter [58]. It has been reported that there is a pore size threshold limit for individual molecules, after which further increases in pore diameter do not enhance the release rate. It has been shown that long-term stability of amorphous drugs in mesoporous channels is provided [59,60]. However, regarding in silica samples with larger pore sizes, increased recrystallization has been observed due to the loss of nano-confinement properties [61,62]. Some authors claim that the drug molecule cannot recrystallize if the confinement space diameter is less than or equal to 15 times the drug molecule diameter [63,64]. To influence drug loading and release, silica particle properties such as particle size and morphology have been examined to a lesser extent due to the difficulties in examining the effect of particle morphology in isolation. Obviously, it is desired to synthesize particles with monodisperse sizes [65,66]. The large specific surface area of mesoporous carriers is especially important to increase the dissolution rate of poorly water-soluble drugs [67]. Increased surface area improves drug loading and its dissolution rates. However, recent studies demonstrated that there is a threshold level after which increasing surface area does not result in a linear increase in drug release rate [68]. This may be explained due to the effective surface area, which is the real parameter that influences the dissolution rate. Further increase in carrier surface area may not lead to further subdivision of the drug because of the presence of increased cohesive forces/surface tension among the drug particles [56].

Structural properties such as pore size and pore volume as well as surface properties of mesoporous matrices can be altered depending on synthesis conditions (time, temperature, precursor type and its concentration) [69]. Furthermore, the silanol groups present at the external surface can be combined with numerous organic moieties containing a variety of functional groups (-NH_2_, -Cl, -CN, -CH_3_, -COOH) to alter their surface characteristics and provide specific drug-carrier interactions [35]. The surface of mesoporous carrier can be functionalized using co-condensation strategy (one-pot synthesis) and grafting method (post-synthesis modification) [35]. The appropriate modification approach can provide desired rate of drug release from the mesoporous carrier. Moreover, surface modification with target-specific ligand facilitates drug transport to the desired site of action, so-called site-specific targeting. Many types of mesoporous silicas like MCM-41 [13], MCM-48 [70], SBA-12 [71], SBA-15 [72], SBA-16 [73], MCF [74] or PHTS [24] were successfully used as carriers in DDSs. Each material is characterized by different structure, morphology and size [27]. In 2001, mesoporous MCM-41 material was used for the first time by Vallet-Regi and co-workers as a carrier for anti-inflammatory drug ibuprofen [13]. This pioneer study initiated the ongoing scientific campaign to search for and use of mesoporous matrices as carriers in drug delivery systems. Over the last twenty years many review papers have been published describing current developments in the usage of mesoporous materials as carriers for various therapeutic agents [8,16,75,76,77,78,79,80,81,82,83,84,85]. Initially, these were simple systems consisting of the mesoporous carrier and the drug molecules. The release of the drugs from as-prepared systems depends mostly on the structure of the mesoporous carrier. Subsequently, the mesoporous surface was modified to create a desired drug-carrier interactions or provide specific target-site delivery of active agent [35]. Nowadays, modern hybrid systems in which the mesoporous matrix is only the element of the complex whole are of interest to many research groups. 

Following the first use of mesoporous silica as a carrier for ibuprofen, this paper will review the latest achievements in the application of various mesoporous materials as the carriers for anti-inflammatory agents. Inflammation is the first response of the human immune system to tissue injury, irritation or infection [86]. Long-term inflammation can result in the damage of tissues or even lead to chronic diseases such as rheumatoid arthritis, asthma and other inflammatory vascular diseases [87,88]. It is also considered a serious risk factor for cancer [89]. Anti-inflammatory agents are often prescribed by doctors or even more often used without the prescription as over the counter (OTC) drugs. However, taking many of them is associated with mild but numerous side effects. This review paper summarizes the achievements in terms of developing mesoporous material-based drug delivery systems for synthetic and natural substances with anti-inflammatory activity.

## 2. Anti-Inflammatory Agents

Pain is a spontaneous sensory sensation and one of the first defence mechanisms [90]. Chronic pain becomes discomfort which subsequently leads to negative changes manifesting, among others, impaired concentration, disordered memorization and even changes in the spinal cord [91]. The consequence is the development of excessive excitability and strengthening pain reactions [92]. Chronic pain can cause anxiety and depression limiting the patient’s functioning in family and society [93]. Hence, it is important to properly treat the pain. Depending on the place of action, two groups of drugs with analgesic effect are distinguished: analgesics of central action and drugs of peripheral action (so-called local anesthetics) [94]. Among analgesics of central action, opioid and non-opioid analgesics are distinguished [95]. The former exhibits numerous side effects primarily on the part of the central nervous system including the depression of the respiratory center [96]. Moreover, opioid use can lead to physical and psychotic addiction [97]. In the treatment of acute and chronic pain, non-opioid analgesics have also been used. Pain therapy always begins with the use of non-opioid drugs. Classic non-opioid anti-pain drugs are aniline derivatives (paracetamol), salicylic acid derivatives (aspirin), derivatives of methylacrylacetic acid (ibuprofen), pyrazolon derivatives (metamizole) and others (e.g., ketorolac) [98]. Drugs from the group of non-steroidal anti-inflammatory drugs (NSAIDS) (salicylic acid derivatives and derivatives of methylacrylacetic acid) exhibit antipyretic and anti-inflammatory modes of action in addition to analgesic effects [99,100]. Among many NSAIDs, a great majority possess a carboxylic function (aspirin, mefenamic acid, indomethacin, diclofenac, ibuprofen). The few exceptions that do not contain this chemical function are phenylbutazone, celecoxib and oxicams (isoxicam, meloxicam, piroxicam) [101]. NSAIDs are commonly used in the treatment of pain, fever and anti-inflammatory diseases [87].

The anti-inflammatory drugs can be administered to the body via different pathways depending on the disease entity and the patient’s condition among others. The administration routes of most common anti-inflammatory agents available on the market are summarized in Table 2.

The use of NSAIDs in the treatment of pain results from the fact that its mediators, mainly prostaglandin PGE_2_, are responsible for pain and swelling in the inflammatory process [102]. The inflammation leads to the release of pro-inflammatory enzymes and cytokines (interleukins 1 and 6; IL-1, IL-6, tumor necrosis factor α; TNF-α) that activate cyclooxygenase (COX) by the cells of the immune system (monocytes) and thus an increase the synthesis of prostanoids [103]. Prostanoids together with histamine and bradykinin released under the influence of PGE_2_ cause an increase in the permeability of blood vessel walls and expand them [104]. In this way, they affect not only the movement of blood cells to the site of inflammation but they are also the mediators of pain to the endings of the sensory nerves. Therefore, inhibition of PGE_2_ synthesis causes a decrease in its concentration, results in anti-inflammatory and analgesic effect. All NSAIDs are inhibitors of COX, an enzyme responsible for the synthesis of prostaglandins [105]. The main side effects resulting from the therapy using NSAIDS are gastric irritation leading to the occurrence of peptic ulcers, irritation of gastrointestinal mucosa, inhibition of platelet aggregation and adverse reactions to the kidneys [86,87,106].

In the therapy of inflammatory bowel disease (e.g., Crohn’s disease, ulcerative colitis), other anti-inflammatory drugs such as mesalazine are necessary [107]. Mesalazine (syn. mesalamine) is a 5-aminosalicylic acid with amine, carboxylic acid and phenolic functions. The substance is rapidly absorbed by the small intestine. It also belongs to the group of COX inhibitors [108]. 

Inflammation is one of the key factors in the development of corneal angiogenesis [109]. The infiltration of inflammatory cells into the injured cornea upregulates multiple mediators, e.g., IL-1, vascular endothelial growth factor (VEGF), TNF-α and prostaglandins which promote the angiogenic process and stimulate the formation of new corneal vessels, opacity and scars [110,111]. To inhibit the inflammation process in the cornea and avoid the systemic side effects, immunosuppressing drugs are locally applied. An example of such drug is cyclosporin A which decreases the transcription activity of activated T cells and thus reduces the inflammation reactions [110]. Probucol is a diphenolic compound capable of reducing the increased activity of COX through the nuclear factor kappa-light-chain-enhancer of activated C cells of the NF-κB pathway. As a result, the reduced expression of pro-inflammatory markers and the decreased disruption of the blood-brain-barrier integrity was observed in vivo. Furthermore, it has been shown that probucol is a potent free radical scavenger protecting the cellular membranes against oxidative damage [110].

Nimesulide is a chemical compound from the group of sulfonamides [112]. It belongs to the semiselective COX-2 inhibitors. Nimesulide is commonly used for pain relieving in painful osteoarthritis disorders and other acute pain states [113,114]. Another drug often used to relief pain and inflammation symptoms in osteoarthritis and rheumatoid arthritis is flurbiprofen [115,116]. The mechanism of action of this phenylalcanoic acid derivative belonging to the group of NSAIDS also involves blockage of the COX-2 enzyme. Flurbiprofen is a non-selective inhibitor of prostaglandin biosynthesis [117]. 

Other group of anti-inflammatory drugs are steroids. Natural glucocorticosteroids are cortisone and hydrocortisone. Glucocorticosteroids obtained synthetically were used to increase the desired activity and reduce negative hormonal effects, primarily from mineralogenic activity [118,119]. Synthetic glucocorticosteroids with representatives such as beclomethasone, betamethasone, budesonide, dexamethasone, flumetasone, methylprednisolone, mometasone, prednisolone, prednisone or triamcinolone are widely used in the treatment of numerous disease entities. Glucocorticosteroids affect basal metabolism [120], immune mechanisms [121] and participate in the body’s reactions to stress [122]. The therapeutic effect of glucocorticosteroids is considered to be mediated by four different mechanisms: classical genomic and secondary non-genomic effects caused by cytosolic glucocorticoid receptors, membrane-bound glucocorticoid receptor-mediated non-genomic effects and non-genomic effects caused by interactions with cellular membranes [123]. Glucocorticosteroids are used in substitution therapy in primary adrenal insufficiency (Addison’s disease) [124], in second adrenal insufficiency [125,126], in congenital adrenal hyperplasia [127] and in the diagnosis of Cushing’s syndrome [128]. Moreover, this group of drugs is used to treat inflammations (rheumatoid arthritis, asthma, liver failure, chronic obstructive pulmonary disease, ulcerative colitis, acute myocarditis, neuroimmunological disorders and inflammatory disease of the skin) [129,130,131,132,133,134,135,136,137]. Anti-allergic properties determine the use of glucocorticosteroids in allergic diseases such as bronchial asthma, allergic dermatoses and allergy to drugs [138]. In turn, the antimitotic effect of this group of drugs is used in the treatment of acute lymphoblastic leukemia [139] and some lymphomas [140]. Immunosuppressive activity is the basis for the use of glucocorticosteroids to abolish immune reactions after tissue and organ transplants [141]. An absolute indication for immediate glucocorticosteroid therapy is life-threatening disease, e.g., anaphylactic shock [142] and edema of the brain [143]. 

Dexamethasone is a glucocorticoid well known to decrease the number of inflammatory cells in airways [144]. It is extensively used for acute lung injury treatment. This inflammatory syndrome is characterized by increased inflammation and severe lung damage. Targeted drug delivery to inflamed lungs has become an attractive research field [145]. Glucocorticosteroids, which are strong anti-inflammatory agents, are commonly used to ameliorate clinical symptoms of acute lung injury. They were shown to limit tissue injury and acute inflammatory response. The dexamethasone therapy decreases the level of proinflammatory cytokines, reduces lung tissue injury and prevents the pulmonary edema [144]. Betamethasone sodium phosphate is the other anti-inflammatory synthetic corticosteroid. This glucocorticosteroid with a strong anti-inflammatory effect was shown to reduce swelling. Thus, it is widely used in various skin dysfunctions like itching, dryness, crusting, redness, scaling or inflammation [146]. 

The most common side effects of long-term non-substitutive therapy with glucocorticosteroids are osteoporosis, stunted growth in children and youth, steroid diabetes, hypertension, hypokalemia, thromboembolism, increased susceptibility to infections, depression/mood changes, poor wound healing, increased risk for infections, moon face, myopathy or skin atrophy [125,147,148]. 

To overcome the side effects of synthetic anti-inflammatory drugs the natural compounds with anti-inflammatory activity are proposed in the therapy of many inflammations and infectious diseases. Natural medicines revealing anti-oxidative, anti-inflammatory, antifungal, antiviral, antibiotic and even anticancer properties are used for thousands of years in various corners of the world as a remedy for many diseases manifested by pain. Intensive studies have been carried out on natural anti-inflammatory agents, like curcumin, with fewer adverse effect. It is a natural polyphenol exhibiting anti-inflammatory, antioxidant and anticancerogenic activity. Curcumin occurs in the rhizomes of *Curcuma longa* [40,108]. Curcumin’s effectiveness in reducing inflammation has been shown to be due to its antiplatelet, antiviral and cytoprotective activity. The main concern limiting medicinal usefulness of curcumin is its low water solubility and bioavailability [86]. Another compound of natural origin demonstrating anti-inflammatory activity is ginsenoside [149]. Ginsenosides (panaxosides) are triterpene saponins. They occur in the plant genus *Panax* and are usually extracted from the root and the stem of the plant. *Panax ginseng* is very popular in Chinese medicine. The genus name *Panax* derives from the Greek and means ‘all-healing’ [149]. Ginsenosides exhibit many biological functions. They possess antiproliferative, neuroprotective, antioxidative, angiogenic and anti-inflammatory properties. Furthermore, it has been demonstrated that ginsenosides increase the activity of osteoblasts, thus leading to enhanced bone tissue healing [150]. Andrographolide is a natural plant-derived diterpene lactone isolated from *Andrographis paniculata* [151]. This plant is widely recognized for its numerous therapeutic properties in India, China, Korea, Japan and Sri Lanka among others [152]. Andrographolide is the main active ingredient of *A. paniculata* giving the plant a bitter taste. It possesses anti-inflammatory, antioxidative, antipyretic, hepatoprotective, antiviral, antithrombotic, anticancer and immunostimulant properties [152]. Andrographolide is commonly used in the treatment of inflammation-related diseases, fever, laryngitis and upper respiratory tract infections in South Asian countries. This diterpene lactone has also found application in cartilage inflammations, osteoarthritis and rheumatoid arthritis therapy. Nevertheless, due to its low aqueous solubility, low bioavailability and high lipophilicity resulting in easy clearance from the place of administration after intra-articular injection, its therapeutic efficiency is negligible [151]. 

## 3. Physicochemical and Biological Characteristics of Mesoporous Material-Based Drug Delivery Systems (DDSs)

The most common mesoporous material used as a carrier in drug delivery systems (DDSs) is mesoporous silica and its more advanced forms such as mesoporous silica nanoparticles, mesoporous silica nanorods or bioactive glasses [153,154,155,156]. Other materials often used for this purpose are mesoporous carbon [157], mesoporous calcium silicate and mesoporous calcium sulfate [150]. The insightful characterization of the physicochemical and biological properties of mesoporous materials is very helpful to confirm the success of the material’s synthesis and in its future bio application. Mesoporous materials are characterized by large pore volume and pore diameter, good mechanical and chemical stability, possibility of surface modification and tunable particle size [35,88]. Moreover, some mesoporous matrices are characterized by biodegradability and biocompatibility [35,158]. In modern DDSs the mesoporous structure modified with wide range of functional groups (organic functions providing drug-carrier interactions, ligands for receptors enabling targeted delivery, ligands providing stimuli-responsiveness) usually act as a reservoir for the drug [154]. DDSs can be characterized using numerous physicochemical methods. Table 3 lists the most common techniques used for physicochemical and biological characterization of mesoporous material-based DDSs.

**Figure 1:**
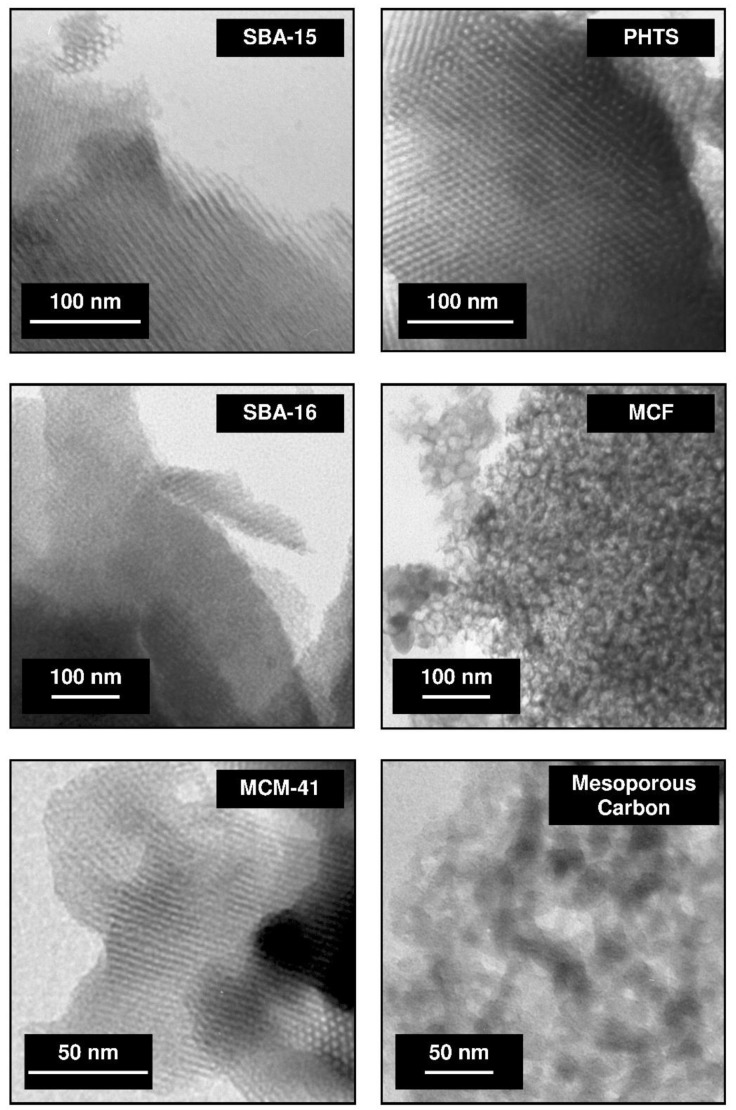
TEM micrographs of various mesoporous materials (abbreviations: SBA-15, 16–Santa Barbara acid; PHTS–plugged hexagonal templated silica; MCF–mesocellular foam; MCM-41–Mobil Composition of Matter). The micrographs were collected using Jeol JEM 1200 EX electron microscope and come from the authors’ own collection.

Mesoporous materials exhibit a large specific surface area of several hundred meters. These matrices include a wide range of materials characterized by different structure, ordering of mesoporous channels and pore geometry [35,69]. In order to obtain the structural information at a nanometer scale resolution for porous materials, transmission electron microscopy (TEM) technique can be used [69]. In Figure 1 the TEM micrographs of well-known mesoporous materials are depicted.

**Figure 2:**
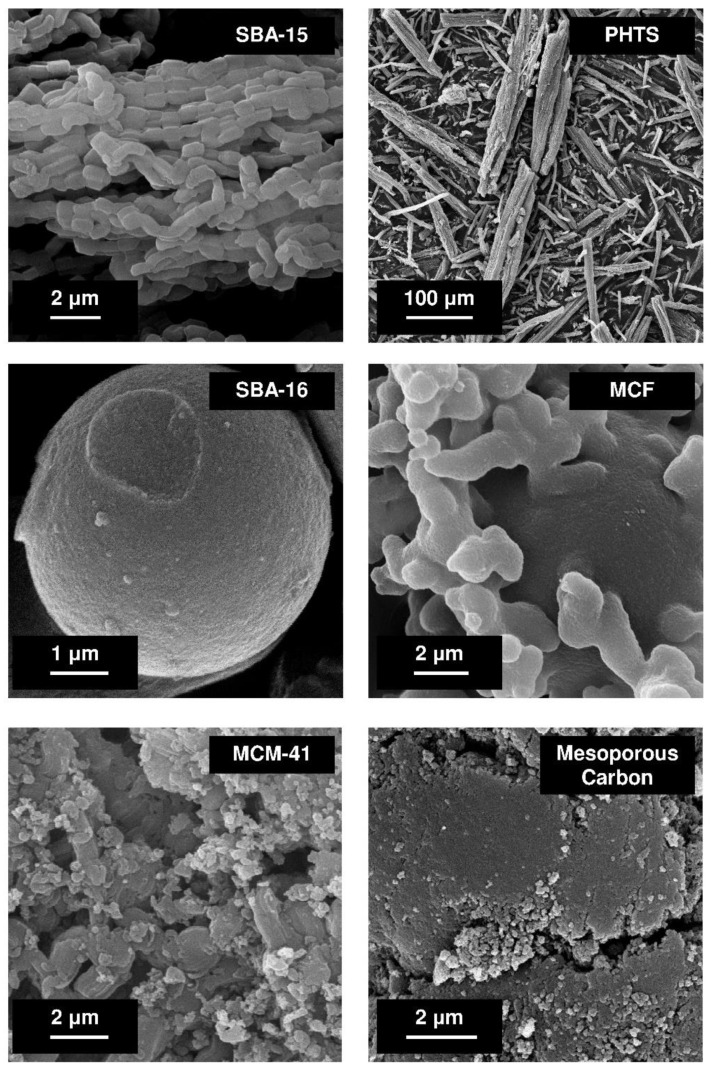
SEM images of various mesoporous materials. The micrographs were collected using Zeiss ELO-40 electron microscope come from the authors’ own collection.

The hexagonal channel arrangement in the structure of SBA-15, PHTS and MCM-41 silicas can be distinguished. SBA-16 silica exhibits the regular structure, MCF matrix reveals the foam-like structure meanwhile mesoporous carbon possesses disordered arrangement. The topography and morphology of mesoporous materials can be studied using scanning electron microscopy [69]. The morphology of SBA-15, PHTS, SBA-16, MCF, MCM-41 silicas and mesoporous carbon are presented in Figure 2.

**Figure 3:**
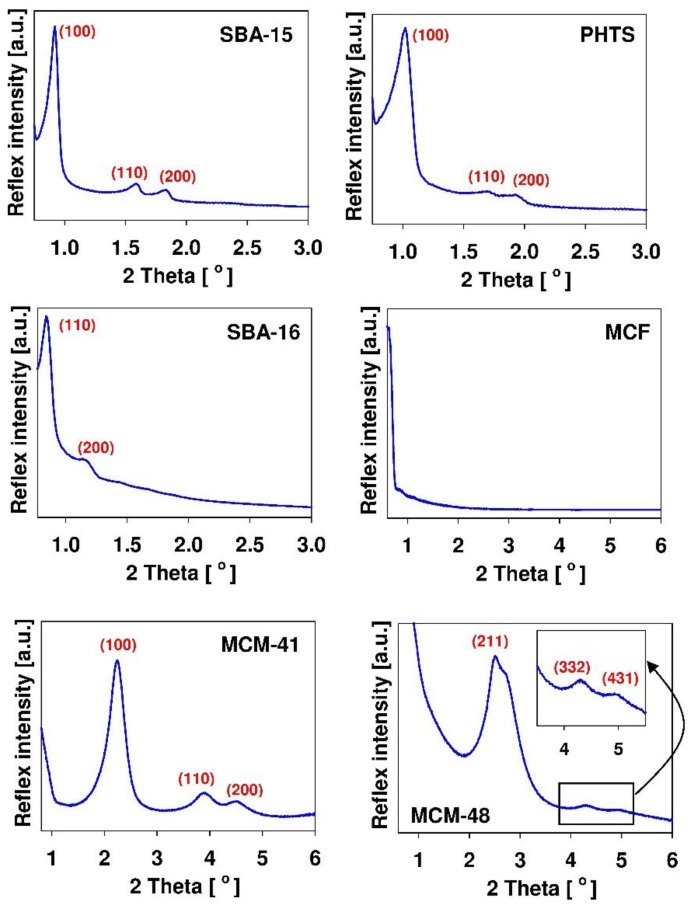
Typical XRD patterns of chosen mesoporous silicas with indices of the diffraction planes. The diffractograms were collected using Bruker D2 PHASER apparatus and come from the authors’ own collection.

SBA-15 material reveals chain-like morphology, PHTS–rough fibers, SBA-16–spherical morphology having diameter of several micrometers, MCF–almost spherical morphology resembling the coral reef, MCM-41 and mesoporous carbon–irregular morphology. The textural characterization of mesoporous solids is usually confirmed by nitrogen adsorption/desorption studies. This technique provides information about micro- and mesoporosity of mesoporous structures. It allows determination of the specific surface area of the carrier, its pore volume and pore diameter as well as the pore geometries of mesoporous channels [69]. By using the powder small angle X-ray diffraction (XRD) method, the ordered structure of mesoporous matrices can be committed. X-ray diffraction is a commonly used technique to evaluate the structure (shape) of the mesoporous molecular sieve. The diffractograms of all ordered nanoporous phases exhibit reflexes in small angle range. Figure 3 demonstrates the typical diffractograms of SBA-15, PHTS, SBA-16, MCF, MCM-41 and MCM-48 silicas.

The presence of reflexes enables the confirmation of mesoporous structure of synthesized materials. Among examined materials, only MCF silica do not exhibit the reflexes. It results from the fact that the large dimensions of the structural motif of MCF material precluded the observation of any X-ray reflexes in the low angle region [165]. Since all mesoporous matrices consist of amorphous silica which exhibit no crystallinity at the atomic level, no reflections can be observed higher than 2θ degrees [69]. Nevertheless, the X-ray diffractogram patterns obtained in the wider range of angles can be useful for confirmation of successful adsorption of the drug within the mesoporous channels [35]. In this case, the amorphous nature of the drug confirms its molecular dispersion on the large surface area of mesoporous carrier. The Fourier transform-infrared (FT-IR) spectroscopy is useful in confirmation of the presence of organic moieties [35]. This technique is based on the fact that each molecule exhibits a specific frequency of internal vibrations that occur in the infrared region of the electromagnetic spectrum [69]. This analytical tool gives information concerning the presence of organic functions at the siliceous carrier surface, thus confirming its successful functionalization. There exist also several other analytical methods used to reveal certain characteristics of mesoporous materials. The size and stability of material can be estimated using the dynamic light scattering (DLS) method. The elemental composition of the surface can be analyzed by using the X-ray photoelectron spectroscopy (XPS) method. The thermal properties can be determined from thermogravimetry analysis (TGA) or differential scanning colorimetry (DSC) analysis whereas the information obtained by solid-state nuclear magnetic resonance (NMR) spectroscopy allows for the study of the local structure of mesoporous matrices. Thus, this technique is complementary to the X-ray diffraction method. The principle of this method is that the number of atoms in the structure of solid samples possess isotopes with nuclear spin, thus, it is possible to observe these isotopes by NMR [69].

The most common techniques used for the characterization of active pharmaceutical ingredients (APIs) loaded in the siliceous matrices are summarized in Table 4.

Advanced DDSs that revolutionized drug delivery studies are sophisticated systems in which the release of the drug depends on many parameters such as the solubility of the drug, its diffusion through the pores and the strength of drug-carrier interactions [35]. The systems are designed to prevent premature drug release (resulting from the usage of so-called gate keepers [16]) and to provide stimuli-responsive drug release using various chemical and physical factors including light, redox state, pH or magnetism [78]. To be used in medicine, these sophisticated materials have to be thoroughly examined both in vitro and in vivo [166]. The toxicity of the systems is usually examined on various cell lines via MTT assay or fluorescence microscopy. Other in vitro experiments include drug loading and release studies, biocompatibility tests, hemolysis assays, biodegradability studies as well as the estimation of anti-inflammatory properties and antibacterial activity (see Table 3). In turn, the in vivo experiments being the final stage of research conducted on living organisms, consist of defining pharmacokinetic and pharmacodynamic properties. The results of these studies allow to determine the therapeutic efficacy (anti-inflammatory response) and biosafety of the systems.

## 4. Mesoporous Material-Based Drug Delivery Systems for Anti-Inflammatory Agents

Among numerous drug delivery platforms gaining attention of the scientific world in the last two decades, mesoporous material-based DDSs exhibit extraordinary properties such as high drug loading capacity, well-defined pore structure, tunable surface chemistry, easily controllable morphology, physical stability and satisfying biocompatibility [30]. Large specific surface area is the main advantageous feature which make mesoporous materials ideal carriers to design multifunctional systems. Mesoporous materials attracted huge scientific attention and several interesting topical reviews are published annually by the leaders in the field, discussing the trends in research and application of these materials as the elements of unique DDSs for use in modern medicine.

Among various porous materials, mesoporous silica and mesoporous carbon have especially gained great attention in recent years due to their superlative properties and structural features. The latter may occur in the form of carbon aerogels which is a special class of lightweight nanoporous materials with tunable porosity and chemical inertness [157]. Mesoporous silica is also the main component of so-called bioactive glass which is an extraordinary class of biomaterials suitable for numerous biomedical applications including wound healing, bone regeneration and cancer therapy [156]. Bioactive glass is composed of silica networks incorporating calcium and is characterized by its mesoporous structure.

Mesoporous silica-based DDSs are especially useful for the incorporation of poorly water-soluble drug molecules. In order to solve the problem of low solubility, many different approaches have been put forward and investigated in depth to improve the dissolution kinetics and bioavailability of poorly water-soluble drugs such as solid dispersions, nanosuspensions, spray drying, cryogenic technologies and prodrug strategies [87,115,155]. It was shown that the problem of low drug solubility can also be overcome by the usage of a carrier substance like mesoporous silica during the preparation of sophisticated drug delivery platforms.

As mentioned before in the first mesoporous silica-based DDS, the siliceous matrix MCM-41 was employed as the carrier for ibuprofen [13]. The sustained drug release from the system was the result of drug molecular dispersion within the pores of the mesoporous carrier. It was shown that mesoporous silica possesses the ability to prevent crystallization of the loaded drug which enhances its dissolution kinetics [13]. Currently, mesoporous silicas used as the elements of modern DDSs provide controlled and intelligent stimuli-triggered drug release. This can be achieved by proper carrier design, including its desired structure and morphology, as well as by sophisticated surface functionalization. Well-defined surface of mesoporous matrices allows conjunction with pore-blocking materials to prevent premature cargo release, with stimuli-responsive ligands (nanovalves or gate keepers [145]) for controlled release or with specific ligands for targeting [151]. Stimuli-responsive drug release refers to the release of the drug trigged by a variety of external factors including temperature, light, pH, heat, tumors, enzyme presence, chiral environment or redox potential [115,161,162]. These approaches were shown to improve the therapeutic efficacy and reduce side effects of the drugs which is of great importance in pharmaceutical practice and can lift DDSs research to a new level.

One outstanding feature resulting from the usage of mesoporous silica as the carrier in modern drug delivery platforms is the enhancement of dissolution kinetics and bioavailability of poorly water-soluble drugs. It is claimed that over 70% of active pharmaceutical ingredients (APIs) belong to the Biopharmaceutical Classification System (BCS) class II and IV [87,115]. The substances from class II are characterized by low solubility and high permeability through biological membranes (intestinal epithelium) meanwhile the components of class IV exhibit both low solubility and low permeability [161]. Thus, the drugs from the BCS Classes II and IV cannot exert satisfactory therapeutic effects due to the insufficient drug concentration in the site of absorption which seriously limits their therapeutic potential. Poor drug solubility in water is associated with the physically stable crystalline nature of most drugs. Thus, it is of great importance to overcome the forces attaching drug molecules within the crystalline lattice [113]. The development of effective DDSs for poorly water-soluble drugs is still a pressing issue for the pharmaceutical industry [157]. By using mesoporous carrier matrices, higher doses of poorly water-soluble drugs can be administered locally, reducing their adverse effects and improving their biodistribution [144].

Numerous studies have been conducted concerning the design of mesoporous material-based DDSs to be used by various administration routes. Mesoporous material-based drug delivery platforms are gaining attention for use through the pulmonary route. The possibility of using mesoporous carrier for dexamethasone in the treatment of airway inflammation was investigated by Gulin-Sarfraz et al. [144]. It was shown that the dissolution kinetics of practically water-insoluble dexamethasone was enhanced when loaded in mesoporous silica particles. Furthermore, drug-loaded particles revealed an increased ability to reach the lower parts of the lungs as compared to free drugs. It was demonstrated that designed delivery platform improved airway distribution and allowed for local treatment. Moreover, the systemic side effects could be reduced, and the possibility of using higher drug doses locally in the lungs is possible. Thus, dexamethasone-loaded mesoporous silica particles revealed its potential as an ideal therapeutic agent for acute airway inflammation [144]. Similar observations were reported by Garcia and colleagues [145]. In this case, the preferential accumulation of mesoporous silica nanoparticles loaded with dexamethasone in inflamed tissues of lungs was ascribed to the vascular nature, high permeability and retention capacity of the lungs. Similar studies were performed by the group of Rosenholm [144] that investigated the feasibility of mesoporous silica particles as carriers for dexamethasone in the treatment of airway inflammation. The particles were administered as aerosol through inhalation to mice models of neutrophil-induced airway inflammation. Down-modulation of the inflammatory response was observed. It has been demonstrated that prepared particles can be employed as corticosteroid carriers in the anti-inflammatory treatment of lung injury [144].

Owing to its convenience, painlessness, easy administration and high patient compliance, oral administration is one from the most accepted and patient preferred drug delivery pathway [155,161]. However, the challenge remains to improve the oral bioavailability of BCS II molecules. Recently, it has been demonstrated that mesoporous material-based DDSs offer advantages for orally administered drugs owing to their ability to deliver the drug to a specific site, avoid digestion in the gastrointestinal tract and control the release and cellular uptake of incorporated drugs [155]. Zhou et al. examined the usage of mesoporous silica nanoparticles as carriers for orally delivered indomethacin [161]. This non-steroidal anti-inflammatory drug exhibits poor water solubility and causes irritation to gastrointestinal mucosa. The carrier was modified with L- and D-tartaric acid. It was shown that as-prepared drug-loaded MSNs revealed higher drug dissolution, oral bioavailability and anti-inflammatory effects compared with naked particles. The superiorities in delivering indomethacin were ascribed to the binding affinity between surface modifying agents and sodium-glucose linked transporter (SLGT). It has been demonstrated that prepared materials triggered chirality of biological environment based on their molecular level chiral function [161]. The group of Aulia examined the anti-inflammatory activity of curcumin-loaded MSNs after oral administration to white male Wistar rats [86]. After oral administration of curcumin-loaded MSNs the anti-inflammatory activity like the one revealed by diclofenac sodium was observed. Furthermore¸ no significant macroscopic and microscopic changes to gastric organs were observed as opposed to the effects caused by diclofenac. The system revealed high potency for medicinal use in inflammation-related diseases.

Mesoporous silica nanoparticles show great potential as delivery platforms in eye therapy due to the possible silicon element supplement to eyes [110]. Sun and colleagues encapsulated the bevacizumab into the pores of MSNs [110]. Separately, cyclosporine A was dissolved in the thermogel. Finally, bevacizumab-loaded MSNs were dispersed in the cyclosporine A-containing thermogel. As-prepared nanohybrid thermogel was administered to the subconjunctiva. It has been demonstrated that the system significantly improved curative treatment in the rabbit model.

Mesoporous materials are an ideal platform for delivery of active agents to be used in the therapy of numerous diseases. Li et al. studied the potential of the application of folic acid-decorated semiconducting polymer dots hybrid mesoporous silica nanoparticles in the therapy of rheumatoid arthritis [154]. The therapeutic effect using the as-prepared delivery nanoplatform was based on the triple photothermal, photodynamic and chemotherapy synergistic treatment. In these studies, mesoporous material served as the reservoir for the hypoxia-activated tirapazamine prodrug. Upon NIR irradiation, the system generated intracellular hyperthermia and excessive singlet oxygen. Local hypoxia caused by molecular oxygen consumption activated the cytotoxicity of tirapazamine. Finally, activated drug killed macrophages inhibited the progression of arthritis. Zheng et al. designed the nanosized pH-responsive DDS for osteoarthritis treatment by using modified mesoporous silica nanoparticles with pH-responsive polyacrylic acid [151]. Prepared nanoplatform was loaded with andrographolide. Compared with pure diterpenoid, andrographolide-loaded nanoparticles revealed enhanced antiarthritic efficacy and chondro-protective capacity as evidenced by lower expression of inflammatory factors and better prevention of proteoglycan loss.

Mahmoudi et al. used smart polymeric nanocomposite based on protonated aluminosilicate-modified mesoporous silica nanoparticles as the carrier for mesalamine in inflammatory bowel disease [108]. The system characterized by high drug loading efficiency was designed to decrease the side effects of this anti-inflammatory drug and to enhance its permeability in intestinal tissues. Drug release studies from as-prepared systems were performed in media simulating conditions occurring in various parts of the gastrointestinal tract. The release experiments indicated increased drug release rate at higher pH simulating the environment of the colon (pH = 8) as compared to the experiments performed in media characterized by lower pH values.

Targeted-lung delivery of dexamethasone using gated mesoporous silica nanoparticles was studied by García-Fernández et al. [145]. Acute lung injury is a critical inflammatory syndrome characterized by high morbidity and mortality. Targeted drug delivery to inflamed lungs is of crucial importance for patients suffering from severe lung damage. The nanodevice based on mesoporous silica nanoparticles loaded with dexamethasone and capped with a peptide targeting the TNFR1 receptor expressed in pro-inflammatory macrophages was designed. It has been demonstrated that after intravenous injection into mice, the nanodevice accumulated in injured lungs. The controlled dexamethasone release resulted in reduction of the levels of TNF-α, IL-6 and IL-1β cytokines [145].

Mesoporous material-based drug delivery systems have a chance to become a valuable tool in the treatment of neuroinflammation. Brain endothelial cells that provide the proper function and integrity of the blood brain barrier are highly susceptible to cellular damage caused by oxidative stress and inflammation. The group of Garcia-Bennet [164] observed the enhanced antioxidant effect of the anti-inflammatory compound probucol when released from mesoporous silica particles. The increased reduction of ROS, COX enzyme activity and PGE_2_ production was measured in human brain endothelial cells treated with probucol-loaded mesoporous silica particles. Prepared system opens new possibilities for rapid suppression of neuroinflammation underlying numerous neurodegenerative diseases.

Mesoporous material-based DDSs offer great promise as co-delivery platforms in the treatment of cancer. The disease has become a major threat to humans in the 21st century [167]. Great progress has been made in cancer therapy and many strategies have been developed to treat the disease. Unfortunately, many traditional therapeutic strategies result in serious adverse effects on normal tissues, making patients suffer from more pain [167]. Other important problem of effective anticancer therapy is the resistance of surviving cells against the employed antineoplastic drugs called multidrug resistance (MDR) [160]. It can be solved by combination therapy consisting in usage of mesoporous material as the carrier for two or even more therapeutic agents. It has been demonstrated that such a system may exert simultaneous and synergistic action on critical metabolic pathways which result in increasing of apoptotic effect via the co-delivery of several drugs [160]. The conjugation of anti-cancer drugs and anti-inflammatory agents has shown promising results [168]. Benova et al. studied the co-delivery approach of 5-fluorouracil anticancer drug in combination with naproxen anti-inflammatory agent using β-cyclodextrin-modified SBA-15 mesoporous silica as a carrier [160]. The system exhibited pH-driven drug release. At physiological pH=7.4 only 16% and 20% of 5-fluorouracil and naproxen were released, respectively. It has been ascribed to the fact that not all pores were capped with β-cyclodextrin molecules. Meanwhile, at acidic pH=5.0 (pH of tumor tissues) nearly 90% of 5-fluorouracil and 99% of naproxen was released due to the unlocking of the pores by β-cyclodextrin [160]. The group of Hu used polypyrrole-coated mesoporous TiO_2_ as a carrier for other pairs of anticancer and anti-inflammatory agents, namely doxorubicin and aspirin prodrugs [167]. It has been demonstrated that under external stimulation of near infra-red and ultrasounds, the system revealed excellent photothermal conversion efficiency and a satisfactory sonodynamic effect. Moreover, simultaneous prodrugs activation and rapid drug release were observed. As a result, a significant tumor inhibition effect obtained through synergistic therapy was achieved. The system was shown to reduce the inflammatory factors such as IL-1β and TNF-α in the blood of mice [167].

The potential of using mesoporous material-based devices as suitable materials for medical and hygienic applications was also reported. Hashemikia et al. grafted betamethasone-loaded SBA-15 mesoporous silica particles on the surface of cotton fabric in order to obtain an antibacterial nanosurface with drug delivery properties [146]. Prepared fabrics exhibited excellent antibacterial activity against Escherichia coli and Staphylococcus aureus even after several washing cycles. Obtained textile surface revealed the potential to be used in wound dressing acting as a gradually releasing corticosteroid reservoir at the affected site for a certain period.

It is also possible to use mesoporous material-based DDSs in bone regeneration [150]. Bone defects are very common in older generations which can be ascribed to osteoporosis among other diseases. Bone scaffolds enhance bone regeneration acting as biological extracellular support for cells. Chen et al. designed a 3D-printed mesoporous calcium silicate/calcium sulfate scaffold loaded with ginsenoside Rb_1_ that provided mechanical stability and promoted cell viability [150]. Experiments revealed that human dental pulp stem cells (hDPSCs) cultivated in ginsenoside-containing scaffold exhibited proliferative ability and higher expression of osteogenic-related proteins and could effectively inhibit inflammation. Very recently, mesoporous bioactive glass nanoparticles containing cerium element were synthesized by Boccaccini et al.as anti-inflammatory and anti-bacterial agents with potential to be used in inflammatory bone diseases and bone infections [156]. Mesoporous bioactive glasses focused the attention of many scientific groups as they found applications in many fields of biomedicine, e.g., as delivery carriers, bioactive fillers and injectable biomaterials. Mesoporous glasses are osteoinductive and they exhibit superior mineralization capability. They were shown to form strong interfacial bonding with both soft and hard tissues. Moreover, similar to other mesoporous matrices, they possess large specific surface area, high pore volume and pore diameter. Depending on their composition and morphology these materials can enhance vascularization and wound healing. It was demonstrated that cerium-incorporated mesoporous glass nanoparticles revealed strong anti-inflammatory activity on lipopolysaccharide-induced RAW 264.7 macrophage cells. Moreover, the antibacterial properties of the material against Staphylococcus aureus and Escherichia coli were demonstrated [156].

Other therapeutic achievements resulting from the usage of mesoporous material-based DDS are briefly described in Table 5.

## 5. Conclusions and Perspectives

This review describes the most recent achievements in using mesoporous materials as the elements of sophisticated drug delivery systems (DDSs). During the past two decades, increasing progress has been made in the synthesis and functionalization of mesoporous matrices to be used as carriers for anti-inflammatory therapeutic agents. The practical utilization of knowledge from the field of material science to biomedical applications offer the promising possibilities for more effective treatment of pain and inflammation-related diseases. Unique properties of mesoporous materials including their large specific surface area, large pore volumes and pore diameters, tunability of the pore sizes, ease of chemical synthesis, possibility of surface functionalization and biocompatibility make these structures very interesting candidates as drug carriers. It is worth to be mentioned that the main advantage of mesoporous matrices is the possibility of their usage to merge different materials and thus to provide various functionalities to the designed system. Mesoporous matrices can act as a basis for the preparation of multifunctional tools for biomedical applications such as promising drug carriers. The outstanding pharmaceutical features resulting from the usage of mesoporous matrices as the carriers of anti-inflammatory agents is the possibility of increasing the dissolution kinetics of drugs that are usually characterized by low water solubility. Moreover, the proper surface functionalization makes mesoporous matrices promising carriers to effectively transport and site-specifically deliver drugs while also minimizing undesired side effects. Mesoporous matrices are superior candidates, especially for the construction of stimuli-responsive drug carriers. In these systems, there is a possibility to control the kinetics of drug release through external stimuli. In this area, huge effort has been made by scientists to develop various gatekeepers that could enable controlled exposure of entrapped drugs upon diverse stimuli. Overall, the research advancements in the use of mesoporous materials as elements of DDSs are exciting and hold great potential for future biomedical applications.

Although mesoporous material-based drug delivery systems are promising tools to be used in the therapy of inflammation, several critical issues regarding their safe use need to be addressed. First, the synthesis and functionalization steps during DDS manufacture have to be reproducible in order to provide valuable material for further in vitro and in vivo testing. Careful analyses of in vitro and in vivo toxicity regarding the chemical composition, size, shape, surface area, porosity, functionalization and charge of the adequate system are needed to facilitate understanding of its behavior in a biological environment. A comprehensive study of biocompatibility, biodegradability and biodistribution of the systems is needed. Although many in vitro and in vivo studies of DDS toxicities are performed, the toxicity studies in the human body are very limited. To summarize, before the introducing the final product to the market, its safety for the human body must be confirmed in detail which is time consuming, tedious and expensive. It seems that the toxicity aspect is the major barrier in the process of DDS commercialization. It should be kept in mind that all components used to fabricate the system have to be biocompatible. The main challenges related to the application of mesoporous material-based DDSs in biomedicine are summarized in Table 6.

## Figures and Tables

**Table 1:** The properties of mesoporous materials influencing the drug loading and release.

Parameter	Drug	Mesoporous Silica	Effect on Drug Loading/Release	Ref.
Pore size, pore geometry	Atorvastatin	Hexagonal SBA-15 silica, MSF with continuous pore system	Significant enhancement in the rapid burst release of drug from both silica (MSF showed a higher degree of burst release) The faster drug release from MSF resulted from bigger pore size of this carrier in comparison to SBA-15 material The 3D spherical pore system and the particular geometry of MSF made the molecular diffusion easier from the inner pores into the release fluid thus avoiding pore blocking	[44]
Pore geometry	Carvedilol	Hexagonal MCM-41 SBA-16 cage-like mesoporous silica	Improved drug dissolution rate for both carriers compared with crystalline form SBA-16 displayed a faster release kinetics which may be attributed to the 3D interconnected pore networks in comparison to 2D cylindrical pores of MCM-41	[45]
Pore size	Furosemide	MCM-41, SBA-15	Fast drug release for both silicas was observed with respect to the crystalline drug (faster for SBA-15) When pores were larger, the diffusion of the drug molecule into the dissolution medium was improved	[46,47]
Pore volume and pore morphology	Ibuprofen	SBA-15 MCM-41 TUD-1	Total pore volume has a significant influence on the degree of drug loading TUD-1 exhibited the fastest drug release due to its highly accessible pore network compared to the undirectional, uniform hexagonal mesopores of SBA-15 and MCM-41	[48]
Particle morphology	Ibuprofen	Irregular and spherical MCM-41 particles	With nearly the same loading of ibuprofen irregularly-shaped particles (with the largest pores) released the drug fastest than spherical particles	[49]
Pore size	Ibuprofen	MSNs (hexagonal MCM-41 and SBA-15, and cubic MCM-48)	Loading capacity in the presence of sufficient ibuprofen was found to be proportional to the pore dimensions (mesopore size SBA-15 > MCM-48 > MCM-41)	[13,50,51]
Pore size and pore geometry	Ibuprofen	MSNs (MCM-41, SBA particles with different pore sizes: cubic SBA-1 with interconnected porosity and micro-/meso-porous hexagonal SBA-3)	Drug loading degree and the release rate was consistent with the decrease in their pore size Interconnected small opening between pore channels in SBA-1 increased the diffusion of encapsulated molecules	[52]
Pore geometry, particle morphology	Ibuprofen	Hexagonal MCM-41 Cubic MCM-48	With the same spherical morphology and particle size but different pore geometry MCM-48 possessing higher surface area and pore volume exhibited significantly larger loading capacity and much quicker drug release than MCM-41	[53]
Pore size	Ibuprofen	MCM-41, SBA-15, SBA-15-LP	The pore size effected the physical state of the drug: drug loaded inside SBA-15-LP (pore size 20 nm) was organized in nanocrystals, drug loaded in MCM-41 and SBA-15 (pore size smaller than 10 nm) was in amorphous state Amorphous drug showed a rapid dissolution while nanocrystalline drug showed a slower dissolution rate	[54]
Pore size	Itraconazole	SBA-15 with pore diameters ranging from 4.5 to 9.0 nm	Broadening the pore size from 4.5 to 6.4 nm greatly improved drug release The supplementary increase in the pore size up to 7.9 and 9.0 nm showed only a minor additional increase	[55]

**Abbreviations:** HMS–hollow mesoporous spheres; MCF–mesocellular foam; MCM-41–Mobile Composition of Matter-41 material; MCM-48–Mobile Composition of Matter-48 material; MSF–mesoporous siliceous foam; MSNs–mesoporous silica nanoparticles; SBA-1–Santa Barbara Amorphous-1 material; SBA-3–Santa Barbara Amorphous-3 material; SBA-15-LP–Santa Barbara Amorphous-15-large-pore material; SBA-16–Santa Barbara Amorphous-16 material; TUD-1–Technical Delft University-1.

**Table 2:** The routes of administration of anti-inflammatory drugs.

Class of Anti-Inflammatory Agents	Drug	Administration Route	Drug Form
NSAIDs	Aceclofenac	Oral	Film-coated tablets
	Acidum salicylicum	Topical	Cream Ointment
	Aspirin	Oral	Effervescent tablets Enteric tablets Granules Modified release tablets Powder Tablets Tablets for the socket
	Bromfenac	Intraocular	Eye drops
	Dexibuprofen	Oral	Film-coated tablets
	Diclofenac	Oral	Enteric tablets Film-coated enteric tablets Film-coated tablets Modified-release and prolonged-release capsules Modified-release and prolonged-release tablets
		Topical	Adhesive bandage Aerosol Gel
	Diethylamine salicylate	Topical	Cream Gel
	Etofenamate	Topical	Aerosol Cream Gel Solution
	Flurbiprofen	Oral	Lozenges Prolonged-release capsules
	Ibuprofen	Intravenous	Solution for injection
		Oral	Capsules Effervescent granules Film-coated tablets Suspension Syrup Tablets
		Rectal	Rectal suppositories
		Topical	Cream Gel
	Indomethacin	Intravenous	Solution for injection
		Oral	Prolonged-release tablets
		Rectal	Rectal capsules Rectal suppositories
		Topical	Ointment
	Ketoprofen	Intravenous	Solution for injection
		Oral	Film-coated tablets Modified-release capsules Modified-release tablets Suspension
		Topical	Gel Spray
		Rectal	Rectal suppositories
	Ketorolac	Intraocular	Eye drops
	Meloxicam	Intravenous	Solution for injection
		Oral	Tablets
	Mefenamic acid	Oral	Tablets
	Metamizole	Intravenous	Solution for injection
		Oral	Granules for oral solution Tablets
		Rectal	Rectal suppositories
	Methyl salicylate	Topical	Cream Ointment
	Naproxen	Oral	Film-coated tablets Enteric tablets Suspension Tablets
		Rectal	Rectal suppositories
		Topical	Gel
	Nepafenac	Intraocular	Eye suspension
	Nimesulide	Oral	Granules/powder for suspension preparation Tablets
	Phenazone	In-ear	Ear drops
	Phenylbutazone	Topical	Ointment
		Rectal	Rectal suppositories
	Piroxicam	Oral	Film-coated tablets Powder for oral solution Tablets
	Tolfenamic acid	Oral	Tablets
Steroids	Beclometasone	Intranasal	Aerosol Suspension
		Pulmonary	Aerosol Solution
	Betamethasone dipropionate	Topical	Cream Ointment Solution
	Betamethasone sodium phosphate	Intramuscular	Suspension for injection
		Intravenous	Solution for injection
		Topical	Cream Ointment
	Budesonide	Intranasal	Aerosol
		Oral	Enteric capsules Prolonged-release capsules
		Pulmonary	Aerosol Powder in capsules for inhalation Powder for inhalation
	Clobetasol propionate	Topical	Cream Ointment Shampoo Solution
	Dexamethasone	Intraocular	Eye drops Eye suspension Ointment
		Oral	Tablets
		Topical	Aerosol
	Fludricortisone acetate	Intraocular/in-ear	Ointment Suspension
		Oral	Tablets
	Hydrocortisone	Intravenous	Powder and solvent for intravenous solution
		Oral	Tablets
		Rectal	Rectal ointment Rectal suppositories
		Topical	Aerosol Cream Ointment
	Mometasone furoate	Oral	Tablets
		Pulmonary	Powder for inhalation
		Topical	Cream Ointment Solution
	Prednisolone	Oral	Tablets
		Topical	Solution

**Table 3:** Physicochemical and biological characterization methods of mesoporous material-based drug delivery systems.

Drug Delivery System (Drug/Carrier)	Physicochemical Characterization Methods	Size/Morphology of Mesoporous Structure	In Vitro Studies	In Vivo Studies	Ref.
Andrographolide/Mesoporous silica nanoparticles modified with pH-responsive polyacrylic acid	TEM DLS FT-IR	100 nm/nanoparticles	Drug release Cytotoxicity test Live/dead cells assay	Histological evaluation Changes in articular cartilage examination	[151]
Aspirin/Cationic polyelectrolyte grafted mesoporous magnetic silica composite particles (magnetic iron oxide Fe_3_O_4_ microparticles modified with SiO_2_ layer and functionalized with vinyl groups) grafted with polyelectrolyte layer composed of isobornyl methacrylate and APTMACl	SEM TEM XRD N_2_ sorption DLS FT-IR XPS TGA	430 nm/spherical particles	Anti-inflammatory activity Thrombolytic activity	---	[159]
Betamethasone sodium phosphate /Cotton fabric with grafted SBA-15 mesoporous silica modified with (3-aminopropyl)triethoxysilane particles) stabilized with chitosan and polysiloxane softener	SEM Stiffness test (bending length) Warp direction (tensile strength) Shirley instrument (air permeability)	1 µm/rope-like morphology	Cytometry Drug release Antibacterial activity	---	[146]
Bevacizumab and cyclosporine A/Silica thermogel nanohybrids (PLGA-PEG-PLGA copolymer and mesoporous silica nanoparticles)	NMR Gel permeation chromatography TEM N_2_ sorption Zeta potential FT-IR	40 nm/nanoparticles	Drug release Cytotoxicity Inhibition effect on corneal neovascularization	Corneal neovascularization effectiveness (biosafety)	[110]
Cerium/Mesoporous bioactive glass nanoparticles	SEM TEM XRD FT-IR N_2_ sorption ICP-OES	100–200 nm/spherical nanoparticles	Anti-inflammatory effect Cytotoxicity Antibacterial activity Biocompatibility	---	[156]
Dexamethasone/Gated mesoporous silica nanoparticles	XRD TEM N_2_ sorption DLS Zeta potential	100 nm/nanoparticles	Biocompatibility (cell viability studies) Inflammatory response	Testing of anti-inflammatory effect Histopathological studies	[145]
Dexamethasone/Mesoporous silica nanoparticles modified with PEG-PEI copolymer (size 1 μm, 200 nm)	N_2_ sorption SEM TEM	200 nm–1 µm/spherical particles	---	Examination of anti-inflammatory response	[144]
5-Fluorouracil and naproxen/System composed of N-(propyl)aniline modified mesoporous silica nanoparticles (SBA-15) and β-cyclodextrin	N_2_ sorption TEM TGA Small-angle XRD microcalorimetry	---	MTT (cell viability) Apoptotic assay	Biocompatibility study (CAM assay, histology)	[160]
Ginsenoside Rb_1_/Mesoporous calcium silicate and calcium sulfate scaffolds	XRD FT-IR SEM	---	Soaking Cell adhesion and proliferation Fluorescent staining ELISA	Implantation of drug-containing scaffold Histological staining	[150]
Ibuprofen/Mesoporous carbon aerogels with different pore sizes (10 nm and 20 nm)	HRTEM N_2_ sorption XRD DSC XPS Zeta potential	---	Release studies (HPLC) Cytotoxicity studies Stability tests	---	[157]
Indomethacin/Mesoporous silica nanoparticles modified with D-tartaric acid and L-tartaric acid	N_2_ sorption FT-IR TEM Zeta potential DSC	200 nm/nanoparticles	Drug release	Anti-inflammation pharmacodynamics Pharmacokinetics study	[161]
Indomethacin/Mesoporous silica nanoparticles modified with TESPSA-L-proline and TESPSA-D-proline	TEM N_2_ sorption FT-IR Circular dichroism XRD TGA DSC Zeta potential	135–252 nm/nanoparticles	Drug release Contact angle measurement Hemolysis assay Cytotoxicity Biodegradability	Bio-adhesion study Gastrointestinal tract retention Distribution pharmacokinetics Anti-inflammatory pharmacodynamics	[162]
Indomethacin/Mesoporous silica nanorods	TEM N_2_ sorption FT-IR Small-angle XRD	---/nanorods, nanoparticles	Cytotoxicity Drug release	Pharmacokinetic studies	[155]
Tirapazamine/Folate acid-decorated semiconducting polymer (PCPDTBT) dots hybrid mesoporous silica nanoparticles	Zeta sizer TEM N_2_ sorption	---/nanoparticles	Photothermal and photodynamic properties Drug loading and NIR-induced release Cytotoxicity Cellular uptake NIR-induced intracellular hypoxia/singlet oxygen detection	Therapeutic efficacy Histological analysis Serum cytotoxicity assay	[154]
Naproxen/Magnetic mesoporous silica nanocomposite (hexagonally ordered mesoporous silica MCM-41 and iron oxide magnetic nanoparticles)	TEM DLS N_2_ sorption FT-IR Magnetic measurements	350 nm × 150 nm/rod-like shape	Cytotoxicity studies (MTT, fluorescence microscopy)	---	[163]
Naproxen sodium salt/MCM-41 mesoporous particles modified with photo-sensitive ligand (cinnamic acid derivative)	IR N_2_ sorption TGA STEM EDX	1 µm/rod-like shape	Drug release	Drug release	[30]
Nimesulide and indomethacin/Carboxyl-functionalized mesoporous silica nanoparticles	FT-IR TEM Small angle XRD N_2_ sorption Zeta potential TGA	100–300 nm/spherical nanoparticles	Drug release	Pharmacokinetic studies (determination of drug concentration in blood) Anti-inflammation pharmacodynamics (evaluation of ankle swelling, measurement of serum TNF-α and IL-1β concentrations histopathological examination)	[87]
Nimesulide/Chiral mesoporous silica nanoparticles with enlarged mesopores	FT-IR Circular dichroism TEM SEM N_2_ sorption	200–300 nm/nanoparticles	Drug dissolution	Pharmacokinetics Anti-inflammatory pharmacodynamics Mucous membrane adhesion	[113]
Probucol/Mesoporous silica particles (AMS-6)	SEM DLS Powder XRD FT-IR N_2_ sorption TGA DSC	4.7 nm/particles	Oxidative stress and cell viability assays DCFDA cellular reactive oxygen stress measurement Mitochondria hydroxyl assay Nitric oxide assay Peroxynitrite assay COX enzyme activity PGE_2_ measurement TNF-α assay Flow cytometry	Measurement of ROS concentration in Zebrafish	[164]
Sulindac/SBA-15 mesoporous silica modified with (3-aminopropyl)triethoxysilane	XRD DSC TEM SEM FT-IR ^1^H-NMR Spectrophotometry	1 µm/rod-like shape	Drug release Cytotoxicity studies	---	[153]

**Abbreviations:** AMS-6–Anionic Mesoporous Silica-6 material. APTMACl–(3-acrylamidopropyl)trimethylammonium chloride. CAM–corioallantoic membrane. COX–cyclooxygenase. DCFDA–2′,7′-dichlorodihydrofluorescein diacetate. DLS–dynamic light scattering. DSC–differential scanning colorimetry. EDX–energy dispersive X-ray spectroscopy. ELISA–enzyme-linked immunosorbent assay. FT-IR–Fourier transform-infrared spectroscopy. HPLC–high performance liquid chromatography. HRTEM–high resolution TEM. ICP-OES–inductively coupled plasma-optical emission spectrometry. IL-1β–interleukin-1β. MCM-41–Mobil Composition of Matter-41. MTT–3-(4,5-dimethylthiazol-2-yl)-2,5-diphenyltetrazolium bromide. NIR – near infrared. NMR–nuclear magnetic resonance. PCPDTBT–poly [2,6-(4,4-bis-(2-ethylhexyl)-4H-cyclopenta [2,1-b;3,4-b]dithiophene)-alt-4,7(2,1,3-benzothiadiazole)]. PEG–polyethylene glycol. PEI–polyethylene imine. PGE_2_–prostaglandin E_2_. PLGA–poly-(DL-lactic acid co-glycolic acid). qRT-PCR–quantitative real-time polymerase chain reaction. SBA-15–Santa Barbara Amorphous-15 material. TEM – transmission electron microscopy. ROS–reactive oxygen species. SEM–scanning electron microscopy. STEM–scanning transmission electron microscopy. TESPSA–(3-triethoxyl-propyl)succinic anhydride. TGA–thermogravimetric analysis. TNF-α–tumor necrosis factor α. XRD–X-ray diffraction. XPS–X-ray photoelectron spectroscopy.

**Table 4:** Methods used for analysis of drugs loaded in mesoporous siliceous matrices.

Characterization Method of Loaded API	Information Obtained
DSC	Confirmation of drug amorphous/crystalline state
Elemental analysis	Assessment of drug content in mesoporous carrier
FT-IR	Confirmation of drug presence in the mesoporous carrier/drug-mesoporous carrier interactions
Low temperature N_2_ sorption	Examination of changes in textural properties of mesoporous carrier after drug loading
NMR	Confirmation of drug-mesoporous carrier interactions
Release profile	Assessment of drug pharmaceutical bioavailability
TGA	Evaluation of drug thermal stability/drug amount in carrier
XRD	Confirmation of drug amorphous/crystalline state

**Abbreviations:** API–active pharmaceutical ingredient. DSC–differential scanning colorimetry. FT-IR–Fourier transform infrared spectroscopy. XRD–X-ray diffraction. NMR–nuclear magnetic resonance. TGA–thermogravimetric analysis.

**Table 5:** Therapeutic achievements resulting from the usage of mesoporous materials-based drug delivery systems (DDSs).

Drug	Carrier	Therapeutic Achievement	Ref.
Aspirin	Cationic polyelectrolyte grafted mesoporous magnetic silica composite particles (magnetic Fe_3_O_4_ microparticles modified with SiO_2_ layer and vinyl groups) grafted with polyelectrolyte layer composed of iBMA and APTMACl	pH-Dependent adsorption and release of anionic drug (through off- and on-capping of polyelectrolyte valve/gate)–controlled drug release in response to specific physiological change Significant thrombolytic activity	[159]
EME	Mesoporous silica nanoparticles modified with amine (-NH_2_) functions	Significant reduction in COX-2 expression EME combined with MSNs showed the therapeutic potential of an anti-inflammatory agent	[88]
Flurbiprofen	Chiral self-assembled mesoporous silica nanoparticles functionalized using L/D-tartaric acid	Enhanced pH-response (carboxyl groups induced stronger electrostatic repulsions between drug and the system) Drug release was inhibited in the chiral environment	[115]
Indomethacin	APTES-functionalized hexagonal mesoporous silica	Increment of drug activity Prevention of the harmful potential of indomethacin on the gastric and hepatic tissues	[168]
Indomethacin	Mesoporous silica nanorods	Excellent dissolution-enhancing effect Better oral bioavailability of drug resulting from ordered helical channels and larger surface area-to-volume ratio of mesoporous silica nanorods	[155]
Indomethacin	MSNs modified with TESPSA-L-proline and TESPSA-D-proline	Both kinds of MSNs significantly improved drug dissolution compared with naked MSNs and exhibited different chiral recognition functions for drug release in the simulated chiral environment in vitro D-MSN could facilitate the drug retention, enhance the in vivo distribution and bioavailability due to the chiral recognition function at the molecular level	[162]
Naproxen	MCM-41 mesoporous silica modified with magnetic Fe_3_O_4_ nanoparticles	After encapsulation of the magnetic nanoparticles into the mesoporous silica the particles keep their superparamagnetic behavior and could be used for vectored drug delivery using magnetic fields and preparation of smart drug delivery systems	[163]
Naproxen	SBA-15 mesoporous silica modified with 3-aminopropyl, phenyl and cyclohexyl groups	Functionalization of the surface with more bulky and more hydrophobic ligands (cyclohexyl, phenyl) led to lower drug loading and release More drug was released in neutral pH in comparison with the acidic pH	[27]
Naproxen sodium salt	MCM-41 mesoporous particles modified with photo-sensitive ligand cinnamic acid derivative	Cinnamic acid derivative molecules located on the surface of MCM-41 served as gatekeepers through which the drug is blocked/released by UV irradiation Cinnamic acid derivative molecules undergo a photo-dimerization reaction by radiation with a wavelength higher than 365 nm, through which the drug molecules were encapsulated in the pores	[30]
Nimesulide	Chiral mesoporous silica nanoparticles with enlarged mesopores	Superior delivery effect (most crystalline drug converted to amorphous phase) Higher oral relative bioavailability and anti-inflammatory effect because enlarge mesopores contributed to load and release more amorphous drug	[113]
Nimesulide Indomethacin	Carboxyl group-functionalized mesoporous silica nanoparticles	Significant improvement of dissolution of drugs due to the beneficial pore structure and pore chemistry Good biocompatibility and bio-adsorption capacity Higher bioavailability of drugs Strong anti-inflammatory effect by delivering more drugs *in vivo*	[87]
Sulindac	APTES-modified SBA-15	Increase of drug dissolution rate Non-toxicity of the system	[153]

**Abbreviations:** APTMACl–(3-acrylamidopropyl)trimethylammonium chloride. APTES–(3-aminopropyl)triethoxysilane. COX–cyclooxygenase. EME-(9S,E)-8-ethyl-9-methylnonadec-6-en-3-one. iBMA–isobornyl methacrylate. MCM-41–Mobil Composition of Matter-41 material. MSNs–mesoporous silica nanoparticles. TESPSA–(3-triethoxyl-propyl)succinic anhydride.

**Table 6:** Challenges related to the application of mesoporous material-based DDSs in biomedicine.

Level	Challenge	Details
Manufacture	Large-scale manufacturing	Protocols for reproducible synthesis and functionalization should be standardized Particles should be stable and dispersible
Bioapplication	Toxicity (acute, chronic)	In vitro In vivo (different animal models, human body)
	Biocompatibility	Animal models
	Biodegradability	Animal models
	Biodistribution	Understanding the interactions between DDS and living organism Accumulation in vital organs causing toxicity (unspecific interactions with non-targeted cells)
	Targeting efficacy	Influence of particle size, surface functionalization, porosity, charge
	Drug delivery	Inertness of DDS during the time needed to reach the target Long-term stability
Introduction to the market	Sophisticated surface modification	Increased cost of the final product
	Commercialization	Safety (short term and long term) of the product must be proven for the human body which is time consuming and laborious
